# The management of healthcare employees’ job satisfaction: optimization analyses from a series of large-scale surveys

**DOI:** 10.1186/s12913-023-09426-3

**Published:** 2023-05-03

**Authors:** Paola Cantarelli, Milena Vainieri, Chiara Seghieri

**Affiliations:** Management and Healthcare Laboratory, Institute of Management and L’EMbeDS, Sant’Anna School of Advanced Studies, Piazza Martiri Della Libertà 33, Pisa, 56127 Italy

**Keywords:** Job satisfaction, Healthcare employees, Healthcare governance, Large-scale viewpoint surveys, Optimization analysis

## Abstract

**Background:**

Measuring employees’ satisfaction with their jobs and working environment have become increasingly common worldwide. Healthcare organizations are not extraneous to the irreversible trend of measuring employee perceptions to boost performance and improve service provision. Considering the multiplicity of aspects associated with job satisfaction, it is important to provide managers with a method for assessing which elements may carry key relevance. Our study identifies the mix of factors that are associated with an improvement of public healthcare professionals’ job satisfaction related to unit, organization, and regional government. Investigating employees’ satisfaction and perception about organizational climate with different governance level seems essential in light of extant evidence showing the interconnection as well as the uniqueness of each governance layer in enhancing or threatening motivation and satisfaction.

**Methods:**

This study investigates the correlates of job satisfaction among 73,441 employees in healthcare regional governments in Italy. Across four cross sectional surveys in different healthcare systems, we use an optimization model to identify the most efficient combination of factors that is associated with an increase in employees’ satisfaction at three levels, namely one’s unit, organization, and regional healthcare system.

**Results:**

Findings show that environmental characteristics, organizational management practices, and team coordination mechanisms correlates with professionals’ satisfaction. Optimization analyses reveal that improving the planning of activities and tasks in the unit, a sense of being part of a team, and supervisor’s managerial competences correlate with a higher satisfaction to work for one’s unit. Improving how managers do their job tend to be associated with more satisfaction to work for the organization.

**Conclusions:**

The study unveils commonalities and differences of personnel administration and management across public healthcare systems and provides insights on the role that several layers of governance have in depicting human resource management strategies.

## Introduction

Measuring employees’ satisfaction with their jobs and working environment have become increasingly common worldwide among government and public organizations across fields, including healthcare [1–4]. Designing personnel policies that fit workers’ perceptions turned out to be uncontroversially relevant. Even more so during challenging times such as those generated by budget cuts and increased demands for public service provision [1] or caused by health and economic emergencies such as the COVID-19 outbreak [5]. The Federal Employee Viewpoint Survey administered by the United States Office of Personnel Management to federal civil servants is just the most famous example of how organizations can monitor workers’ attitudes and perceptions to manage human capital effectively [6, 7]. Among the OECD governments administering surveys to their employees, the most common focus is on job satisfaction. Indeed, the number of countries that center the items of questionnaires on employees’ satisfaction is larger than those centering on work-life balance, employee motivation, or management effectiveness [1].

Public healthcare organizations are not extraneous to the irreversible trend of measuring employee perceptions to boost performance and improve service provision [8–11]. Indeed, asking employees to express their opinion on the work environment in which they operate daily to provide social and health services to citizens make them involved in the management and planning of activities. At the same time, employees’ feedback become a valuable resource for organizational management and an important tool to initiate targeted, efficient and effective improvement processes based on staff needs and expectations. Considering the multiplicity of aspects associated with job satisfaction, it is important to provide management with a method for assessing which elements it may be useful to focus on.

Our study is dedicated to identifying the mix of factors that are associated with an improvement of health professionals’ job satisfaction related to unit, organization, and regional government in the context of a series of large-scale surveys. Investigating employees’ satisfaction and perception about organizational climate with different governance levels seems essential in light of extant evidence showing the interconnection as well as the uniqueness of each governance layer in enhancing or threatening motivation and satisfaction across public administration fields, including government [12, 13] and healthcare [14–16].

Our work provides several contributions to existing knowledge on the correlates of job satisfaction among civil servants in health organizations. Our findings may prove useful to scholars and practitioners alike. Firstly, to the best of our knowledge, this study is one of the first that employs optimization models for this purpose. In doing so, we espouse recent invitations to develop research projects that are context-sensitive and practical so to be able to develop middle range theories because optimization analyses is primarily meant to speak to managers and healthcare professionals [17–19]. Indeed, the main objective of the calculation of the optimization function is to provide some indications with a managerial value on the most efficient group of predictors – organizational variables – that can drive a preset level of improvement in job satisfaction so to close the science-practice gap in healthcare management work. In other words, the calculation provides a numerical information that shows how much organizational aspects weigh on the level of satisfaction. It was introduced for the first time in the field of health performance analysis by a group of researchers from the Ontario Ministry of Health in Canada [20, 21] and subsequently used in the Italian context to analyze patient satisfaction emergency departments and nursing homes [22, 23]. The use of optimization techniques in public administration is largely unexplored at the moment. Secondly, although unable to collect data across healthcare systems in the world, we account for common critiques about the external validity of findings in public administration research by combining large samples and survey replications in our research design [24, 25]. Even in the country where the study is set, the number of respondents in our work is rather unique.

## Job satisfaction in mission-driven organizations: a literature overview

Job satisfaction is one of the most investigated constructs by practitioners and scholars alike across disciplines such as health services [2, 26], public administration [27] and applied psychology [28, 29]. In the words of Hal Rainey [30], “thousands of studies and dozens of different questionnaire measures have made job satisfaction one of the most intensively studied variables in organizational research, if not the most studied” (p. 298).

Scholars across fields such as public administration, mainstream management, and psychology agree that work satisfaction construct includes facets related to the fulfillment of various and evolving individual needs and to the fit with numerous and changing organizational level variables [28]. Recent definitions by public administration and management scholars portray job satisfaction as an “affective or emotional response toward various components of one’s job” [31] (p. 246) or as “how an individual feels about his or her job and various aspects of it usually in the sense of how favorable – how positive or negative – those feelings are” [30] (p. 298). Previous definitions in mainstream management and applied psychology describe job satisfaction as “the feelings a worker has about his job” [32] (p. 100) or as “a pleasurable or positive emotional state, resulting from the appraisal of one’s job or job experiences” [33] (p. 1304).

The breadth and depth of scholarship onto job satisfactions has nurtured efforts to synthesize and systematize available knowledge through meta-analyses and systematic literature reviews in recent years. For instance, Cantarelli and colleagues [27], collected quantitative information from primary studies published in 42 public administration and management journals since 1969 and performed a meta-analysis of the relationships between job satisfaction and 43 correlates, which span from mission valence, job design features, work motivation, person job-fit, and demographic characteristics. Furthermore, Vigan and Giauque [34] present results from a systematic review of the association between work environment attributes, personal characteristics, and work features on the job satisfaction of public employees in African countries. Then, meta-analytic findings show a positive correlation between job satisfaction and public service motivation [35] and pay satisfaction [36].

At the same time, novel studies on work satisfaction among employees across typologies of organizations do not seem to have come to an end. To the contrary, for example, observational work still investigate the individual and organizational correlates of employees’ satisfaction in public healthcare organizations [3, 16, 37–42] and government institutions more in general [7, 43, 44]. A similar interest pertains to employees’ preferences in experimental scholarship in public hospital [45] and public organizations [46].

Based on the evidence summarized above, we investigate the association between public employees’ job satisfaction to work for their unit, organization, and government system and variables that pertains to the following broad domains: workplace safety, human resource management practices at the team level, supervisors’ managerial capabilities, management practices at the organizational level, and training opportunities.

## Methods

Building on such experiences as the Federal Employees Viewpoint Survey [47] and the NHS staff survey, several healthcare systems in Italian Regions administer organizational climate questionnaires to all employees on a routine basis thanks to their collaboration with the Management and Healthcare Laboratory (Scuola Superiore Sant’Anna, Pisa, Italy). Italy currently features a national health service with three main hierarchical levels. The first layer is that of the national health departments that define general strategies, laws, and regulations and set general targets. Regional governments, then, are the second hierarchical level. They are in charge of implementing such strategies and meeting such targets. The 21 Italian Regions are autonomous in this implementation phase. As a result, the variation in the governance structures and healthcare services is large among regional health systems. The third layer includes all organizations (i.e., local health authorities, hospitals, and teaching hospitals) that are at the front-line of health services provision to the population.

The decision to administer an organizational climate survey pertains to the regional government. Members of the Management and Healthcare Laboratory (authors included) design organizational climate surveys together with regional healthcare systems, which, at its core, are interested in using results for sustaining managerial change across the healthcare system. The rationale behind the analysis of region-wide data is multifaceted. First of all, the measurement instrument used in our study has been validated [48, 49] and used in previous work [9] for data analysis at the regional and subsequently organizational level. Secondly, our presentation of results tends to score high on ecological validity because of the mechanisms that govern the provision of healthcare services in Italy where decisions taken at the regional level are binding for organizations within the region. Thirdly, the presentation of results by region resonates with well-established practices on the international stage. Just as an example, NHS staff results are presented at the national level also. As a consequence, our survey includes management variables—such as communication, information sharing, training, budget procedures – that tend to cross the borders of professions. The participation of healthcare employees to the questionnaire is voluntary and anonymous. The survey is composed of statements to which respondents indicate their level of agreement on a 1 to 5 Likert-type sale (1 means full disagreement and 5 full agreement). The questionnaire measures employees’ perceptions about their job, organization, management practices, communication and information sharing processes, training opportunities, budget system, and working conditions [9, 50].

The outcome variables in this study relate to employees’ job satisfaction for three hierarchical levels, namely satisfaction with one’ unit, organization, and regional health service. These layers are key in the Italian healthcare system. In fact, all three levels hold levers that can be pulled to affect job satisfaction. In particular, we used the following statements:

- I am satisfied to work in my unit.
- I am proud to tell others that I work in this organization.
- I am proud to work for the health service of my Region.

We regress each of these three outcome variables on the following list of correlates, which are survey items that tap into different theoretical domains and represent dimensions that can be modified through organizational change initiatives:

- The equipment in my unit is adequate.
- My workplace is safe (electrical systems, fire and emergency measures, etc.).
- My workload is manageable.
- Meetings are organized regularly in my unit.
- Work is well planned in my unit and this allows us to achieve goals.
- Periodically I am given feedback from my supervisor on the quality of my work and the results achieved.
- My suggestions for improvement are considered by my supervisor.
- I feel like I'm part of a team that works together to achieve common goals.
- My supervisor knows how to handle conflict.
- I agree with the criteria adopted by my supervisor to evaluate my work.
- My supervisor is fair in managing subordinates.
- I believe that my supervisor carries out his job well.
- My organization encourages change and innovation.
- The organization encourages information sharing.
- My supervisor encourages information sharing.
- I know annual organizational goals.
- I know annual organizational accomplishments.
- The training activities offered by my organizations are useful in enhancing my competences.
- The training activities offered by my organizations are useful in improving my communication skills with colleagues.
- I appreciate how managers manage the organization.
- My organization stimulates me to give my best in my work.
- I am motivated to achieve organizational goals.
- In my organization, merit is a fundamental value.
- In my organization, the professional contribution of everyone is adequately recognized.

Following the methodology of Brown and colleagues [20], the first phase for the calculation of the optimization function consists of an ordinal logistic regression in which satisfaction is predicted by the organizational variables of interest listed above. The second phase, then, combines the regression coefficients with the average values ​​of the items of interest to identify, under certain pre-established mathematical constraints, the set of organizational variables that, with a certain improvement (always less than 15% for constraints required by the type of analysis) allows to reach a fixed level of overall satisfaction. Thus, optimization techniques allow the identification of the most efficient mix of predictors of employees’ satisfaction to help guide improvement efforts. An important information to consider when reading the results of these types of surveys is that improving the score of a variable that is very close to its benchmark is more difficult than that of a variable that is far from it. It is important to underline that the model is built on the average of the answers, so it does not refer to the strategies to be adopted in cases of falling perceptions related to the organizational climate. In other words, the model does not focus on ways to recover the satisfaction of particularly unsatisfied staff. As for the second phase of the statistical analysis, we used a 5 percent improvement of the job satisfaction outcome variables.

The two phases of analysis listed above have been repeated for each of the four Regions that are included in this study. Region A administered the organizational survey in April and May 2018, Region B in December 2018 and January 2019, Region C in March and April 2019, and Region D between mid-October and mid-December 2019. Respondents are 73,441 healthcare employees, of which 24,869 work in Region A; 5,078 in Region B; 21,272 in Region C; and 22,222 in Region D. The response rates are as follows: 28 percent for Region A, 27 percent for Region B, 39 percent for Region C, and 45 percent for Region D.

## Results

Table 1 presents the demographic characteristics of respondents for each of the four healthcare systems included in our study. In all four cases separately, the average age of participants is not significantly different from the corresponding regional average of all healthcare professionals. Female professionals are slightly overrepresented in all regions compared to the national average of female healthcare professionals. The distribution of respondents across job families in each of the four samples is comparable to the national distribution of healthcare employees [51].

**Table 1:** Demographic characteristics of respondents for each of the four healthcare systems included in our study

	**Region A**	**Region B**	**Region C**	**Region D**
**Number of respondents**	24,869	5,078	21,272	22,222
**Average age in years (standard deviation)**	49.21 (8.77)	47.53 (9.61)	47.74 (9.63)	49.05 (9.14)
**Female (percentage)**	*73*	*76*	*75*	*74*
**Professional family (percentage)**				
Medical doctors	*15*	*13*	*13*	*16*
Nurses	*38*	*41*	*43*	*44*
Nursing assistants	*15*	*16*	*15*	*12*
Technicians	*12*	*14*	*15*	*13*
Administrative staff	*16*	*11*	*12*	*9*
N/A	*4*	*5*	*2*	*6*

Table 2 displays the average job satisfaction, by regional healthcare system and by governance level – namely unit, organization, and Region – along with average standard deviation in parenthesis. Overall, the satisfaction to serve one’s organization is lower than the satisfaction to work for the unit and the regional healthcare system.

**Table 2:** Job satisfaction average and standard deviation, by regional healthcare system, by governance level

	**I am satisfied to work in my unit**		**I am proud to tell others that I work in this organization**		**I am proud to work for the health service of my Region**	
	**Average**	**Standard deviation**	**Average**	**Standard deviation**	**Average**	**Standard deviation**
**Region A**	3.27	1.19	2.90	1.22	3.26	1.15
**Region B**	3.38	1.19	3.02	1.19	3.41	1.12
**Region C**	3.29	1.22	2.96	1.23	3.38	1.17
**Region D**	3.42	1.20	3.08	1.21	3.49	1.14

Table 3 presents the results of the logistic regression on the satisfaction to work for one’s unit across regional healthcare systems. In all regions, keeping everything else constant, professionals’ satisfaction to serve their unit is strongly and positively associated with the following constructs: adequate equipment, work safety, manageable workload, well-planned work, consideration of one’s improvement proposals, sense of being part of a team, agreement with the criteria for individual performance assessment, appreciation for the competences of one’s supervisor, organizational stimulation to give one’s best, and motivation to achieve organizational goals. All relationships are statistically significant at the 0.01 level. In Region A, coeteris paribus, training activities to enhance one’s competences and appreciation for how managers manage the organization are also positively related to job satisfaction at the unit level (*p* < *0.001* and *p* = *0.001*, respectively). As for Region B, keeping everything else the same, supervisor’s fairness in managing subordinates and training opportunities are additional positive correlates (*p* = *0.003* and *p* = *0.004*, respectively). In Region C, everything else equal, the following items also correlates positively with the outcome: supervisor’s fairness in managing subordinates (*p* < *0.001*), supervisors’ encouragement of information sharing (*p* = *0.018*), training opportunities to improve one’s skills (*p* < *0.001*), and appreciation for how managers manage the organization (*p* = *0.033*). Lastly, in Region D, everything else equal, the additional positive correlates of job satisfaction are the following: supervisor’s ability to fairly treat subordinates (*p* < *0.001*), training opportunity to improve professional competences (*p* < *0.001*), and appreciations for managers (*p* < *0.001*).

**Table 3:** Logistic regression results for employees’ satisfaction to work for their unit

	**Region A**				**Region B**				**Region C**				**Region D**			
	**b**	**SE**	**z**	***p***	**b**	**SE**	**z**	***p***	**b**	**SE**	**z**	***p***	**b**	**SE**	**z**	***p***
The equipment in my unit is adequate	0.16	0.01	10.92	*0.000*	0.09	0.03	2.72	*0.006*	0.09	0.02	6.18	*0.000*	0.11	0.01	7.79	*0.000*
My workplace is safe (electrical systems, fire and emergency measures, etc.)	0.11	0.01	7.99	*0.000*	0.06	0.03	1.98	*0.029*	0.08	0.01	5.55	*0.000*	0.10	0.01	7.41	*0.000*
My workload is manageable	0.23	0.01	18.60	*0.000*	0.24	0.03	9.08	*0.000*	0.26	0.01	19.64	*0.000*	0.25	0.01	19.21	*0.000*
Meetings are organized regularly in my unit	-0.03	0.01	-2.79	*0.995*	-0.03	0.03	-1.29	*0.896*	-0.09	0.01	-6.50	*1.000*	-0.01	0.01	-0.43	*0.664*
Work is well planned in my unit and this allows us to achieve goals	0.28	0.02	16.47	*0.000*	0.32	0.04	8.52	*0.000*	0.37	0.02	20.45	*0.000*	0.35	0.02	18.83	*0.000*
Periodically I am given feedback from my supervisor on the quality of my work and the results achieved	-0.05	0.02	-3.54	*0.999*	-0.06	0.03	-1.65	*0.944*	-0.07	0.02	-4.36	*1.000*	-0.05	0.02	-3.16	*0.998*
My suggestions for improvement are considered by my supervisor	0.26	0.02	15.74	*0.000*	0.22	0.04	5.88	*0.000*	0.19	0.02	10.62	*0.000*	0.15	0.02	8.29	*0.000*
I feel like I'm part of a team that works together to achieve common goals	0.75	0.02	45.53	*0.000*	0.81	0.04	22.21	*0.000*	0.71	0.02	39.66	*0.000*	0.78	0.02	43.72	*0.000*
My supervisor knows how to handle conflict	-0.18	0.02	-9.23	*1.000*	-0.17	0.04	-3.92	*1.000*	-0.13	0.02	-6.26	*1.000*	-0.11	0.02	-5.39	*1.000*
I agree with the criteria adopted by my supervisor to evaluate my work	0.20	0.02	10.05	*0.000*	0.19	0.04	4.53	*0.000*	0.21	0.02	10.08	*0.000*	0.17	0.02	8.18	*0.000*
My supervisor is fair in managing subordinates	0.02	0.02	1.07	*0.147*	0.12	0.04	2.98	*0.003*	0.19	0.02	9.70	*0.000*	0.18	0.02	9.03	*0.000*
I believe that my supervisor carries out his job well	0.53	0.02	24.28	*0.000*	0.47	0.05	9.79	*0.000*	0.44	0.02	19.12	*0.000*	0.50	0.02	21.24	*0.000*
My organization encourages change and innovation	-0.06	0.02	-3.61	*0.999*	0.01	0.04	0.18	*0.428*	-0.01	0.02	-0.57	*0.714*	-0.07	0.02	-3.70	*1.000*
The organization encourages information sharing	-0.01	0.02	-0.42	*0.662*	-0.03	0.04	-0.75	*0.772*	-0.03	0.02	-1.94	*0.968*	-0.10	0.02	-5.59	*1.000*
My supervisor encourages information sharing	0.02	0.02	0.89	*0.191*	0.02	0.04	0.62	*0.269*	0.04	0.02	2.22	*0.018*	-0.02	0.02	-1.07	*0.852*
I know annual organizational goals	0.00	0.02	-0.22	*0.587*	-0.07	0.05	-1.54	*0.932*	0.00	0.02	-0.05	*0.518*	-0.04	0.02	-1.93	*0.968*
I know annual organizational accomplishments	-0.06	0.02	-3.59	*0.999*	-0.03	0.05	-0.68	*0.748*	-0.05	0.02	-2.03	*0.974*	-0.05	0.02	-2.20	*0.982*
The training activities offered by my organizations are useful in enhancing my competences	0.12	0.02	8.20	*0.000*	0.09	0.03	2.87	*0.004*	0.09	0.02	5.87	*0.000*	0.08	0.02	5.29	*0.000*
The training activities offered by my organizations are useful in improving my communication skills with colleagues	0.02	0.01	1.56	*0.065*	0.03	0.02	1.13	*0.133*	0.00	0.01	-0.34	*0.633*	0.01	0.01	0.56	*0.289*
I appreciate how managers manage the organization	0.06	0.02	3.30	*0.001*	0.03	0.04	0.63	*0.268*	0.04	0.02	1.91	*0.033*	0.11	0.02	5.33	*0.000*
My organization stimulates me to give my best in my work	0.31	0.02	13.08	*0.000*	0.29	0.05	5.66	*0.000*	0.33	0.02	13.46	*0.000*	0.32	0.03	12.75	*0.000*
I am motivated to achieve organizational goals	0.22	0.02	11.82	*0.000*	0.27	0.04	6.47	*0.000*	0.23	0.02	11.26	*0.000*	0.23	0.02	11.04	*0.000*
In my organization, merit is a fundamental value	-0.13	0.02	-5.14	*1.000*	-0.07	0.05	-1.26	*0.891*	-0.06	0.03	-2.24	*0.983*	-0.09	0.03	-3.41	*0.999*
In my organization, the professional contribution of everyone is adequately recognized	-0.06	0.02	-2.36	*0.987*	-0.10	0.05	-1.79	*0.958*	-0.13	0.03	-5.20	*1.000*	-0.08	0.03	-2.99	*0.997*
theta_0	4.08	0.06	63.48	*0.000*	4.12	0.16	26.52	*0.000*	4.21	0.07	60.47	*0.000*	4.13	0.07	59.52	*0.000*
theta_1	6.15	0.07	88.83	*0.000*	6.06	0.16	36.95	*0.000*	6.11	0.07	81.71	*0.000*	6.09	0.07	82.52	*0.000*
theta_2	8.60	0.08	107.97	*0.000*	8.54	0.19	45.94	*0.000*	8.53	0.09	99.50	*0.000*	8.64	0.09	101.35	*0.000*
theta_3	11.37	0.09	122.45	*0.000*	11.31	0.21	52.94	*0.000*	11.31	0.10	112.79	*0.000*	11.36	0.10	114.56	*0.000*

Table 4 sows the results of the optimization function, set for a 5 percent improvement in average value of the item “I am satisfied to work in my unit.” Predictions tend to be consistent across regional healthcare systems. In all regions, in fact, keeping everything else the same, the job satisfaction improvement at the unit level is associated with an improvement in the mean value of the following constructs: well-planned work in the team, perception of being part of a team that work towards shared goals, and perception that the supervisor can carry out the job well. More precisely, the percentages of improvement for these three correlates are as follows, respectively: 1, 15, and 12 for Region A, 1, 15, and 14 for Region B; 7, 15, and 13 for Region C; and 2, 15, and 13 for Region D.

**Table 4:** Optimization function for employees’ satisfaction to work for their unit (+ 5 percent in the mean value)

	**Region A**	**Region B**	**Region C**	**Region D**
The equipment in my unit is adequate				
My workplace is safe (electrical systems, fire and emergency measures, etc.)				
My workload is manageable				
Meetings are organized regularly in my unit				
Work is well planned in my unit and this allows us to achieve goals	1%	1%	7%	2%
Periodically I am given feedback from my supervisor on the quality of my work and the results achieved				
My suggestions for improvement are considered by my supervisor				
I feel like I'm part of a team that works together to achieve common goals	15%	15%	15%	15%
My supervisor knows how to handle conflict				
I agree with the criteria adopted by my supervisor to evaluate my work				
My supervisor is fair in managing subordinates				
I believe that my supervisor carries out his job well	12%	14%	13%	13%
My organization encourages change and innovation				
The organization encourages information sharing				
My supervisor encourages information sharing				
I know annual organizational goals				
I know annual organizational accomplishments				
The training activities offered by my organizations are useful in enhancing my competences				
The training activities offered by my organizations are useful in improving my communication skills with colleagues				
I appreciate how managers manage the organization				
My organization stimulates me to give my best in my work				
I am motivated to achieve organizational goals				
In my organization, merit is a fundamental value				
In my organization, the professional contribution of everyone is adequately recognized				

Table 5 displays the logistic regression results for professionals’ satisfaction to work for their organization. In all regions, everything else equal, the positive correlates at the 0.05 or smaller significance level are the following: adequate equipment, workplace safety, sense of being part of a team, supervisor’ abilities to do a good job, training opportunities to enhance competences, appreciation for how managers manage the organization, organizational stimuli to give one’s best on the job, and motivation to achieve organizational goals. The relationship between the satisfaction to work for one’s organization and the degree to which one’s work is manageable is positive at the 0.05 significance level for all regions except Region A, everything else constant. Having a well-planned work is a significant correlate in Region D only (*p* = *0.020*), ceteris paribus. Participants’ perceptions that their suggestions for improvement are taken into consideration are significantly related to satisfaction in Regions A and B only, keeping everything else constant (*p* = *0.001* and *p* = *0.043* respectively). Region C is the only that displays an association between the outcome of interest and respondents’ agreement with the criteria adopted to evaluate individual performance, ceteris paribus (*p* = *0.025*). Further, everything else equal, job satisfaction to work for one’s organization is positively associated with the degree to which the organization encourages change and innovation in Region A (*p* < *0.001*), in Region C (*p* = *0.003*), and Region D (*p* < *0.001*). Lastly, respondents in Region A and D show a significant association between the outcome and what supervisors do to encourage information sharing, everything else kept constant (*p* = *0.041*, *p* = *0.038*, and *p* = *0.038*, respectively).

**Table 5:** Logistic regression results for employees’ satisfaction to work for their organization

	**Region A**				**Region B**				**Region C**				**Region D**			
	**b**	**SE**	**z**	***p***	**b**	**SE**	**z**	***p***	**b**	**SE**	**z**	***p***	**b**	**SE**	**z**	***p***
The equipment in my unit is adequate	0.10	0.01	6.76	*0.000*	0.07	0.03	2.20	*0.018*	0.06	0.02	4.18	*0.000*	0.04	0.02	2.38	*0.012*
My workplace is safe (electrical systems, fire and emergency measures, etc.)	0.14	0.01	10.08	*0.000*	0.11	0.03	3.72	*0.001*	0.11	0.01	7.57	*0.000*	0.08	0.01	5.71	*0.000*
My workload is manageable	0.02	0.01	1.65	*0.055*	0.06	0.03	2.37	*0.013*	0.05	0.01	3.58	*0.001*	0.05	0.01	4.01	*0.000*
Meetings are organized regularly in my unit	-0.03	0.01	-2.04	*0.975*	-0.02	0.03	-0.57	*0.713*	-0.02	0.01	-1.47	*0.924*	-0.07	0.01	-4.90	*1.000*
Work is well planned in my unit and this allows us to achieve goals	0.00	0.02	0.23	*0.411*	0.06	0.04	1.54	*0.068*	-0.01	0.02	-0.41	*0.658*	0.04	0.02	2.15	*0.020*
Periodically I am given feedback from my supervisor on the quality of my work and the results achieved	-0.05	0.02	-2.94	*0.997*	-0.06	0.03	-1.73	*0.952*	-0.10	0.02	-6.09	*1.000*	-0.11	0.02	-6.18	*1.000*
My suggestions for improvement are considered by my supervisor	0.06	0.02	3.52	*0.001*	0.07	0.04	1.78	*0.043*	0.02	0.02	1.03	*0.156*	0.02	0.02	0.82	*0.210*
I feel like I'm part of a team that works together to achieve common goals	0.22	0.02	13.73	*0.000*	0.19	0.04	5.45	*0.000*	0.20	0.02	11.53	*0.000*	0.20	0.02	11.27	*0.000*
My supervisor knows how to handle conflict	-0.12	0.02	-6.40	*1.000*	-0.10	0.04	-2.39	*0.988*	-0.08	0.02	-4.05	*1.000*	-0.09	0.02	-4.34	*1.000*
I agree with the criteria adopted by my supervisor to evaluate my work	0.01	0.02	0.69	*0.249*	0.00	0.04	-0.10	*0.541*	0.04	0.02	2.05	*0.025*	0.00	0.02	0.02	*0.491*
My supervisor is fair in managing subordinates	-0.06	0.02	-3.21	*0.998*	-0.09	0.04	-2.23	*0.983*	-0.02	0.02	-0.97	*0.829*	-0.03	0.02	-1.25	*0.889*
I believe that my supervisor carries out his job well	0.17	0.02	7.93	*0.000*	0.15	0.05	3.07	*0.002*	0.15	0.02	6.69	*0.000*	0.19	0.02	7.93	*0.000*
My organization encourages change and innovation	0.10	0.02	5.46	*0.000*	0.03	0.04	0.67	*0.254*	0.06	0.02	2.98	*0.003*	0.10	0.02	5.36	*0.000*
The organization encourages information sharing	-0.04	0.02	-2.35	*0.987*	0.01	0.04	0.21	*0.417*	-0.04	0.02	-2.30	*0.985*	-0.01	0.02	-0.78	*0.779*
My supervisor encourages information sharing	0.03	0.02	1.80	*0.041*	0.05	0.04	1.19	*0.122*	0.03	0.02	1.60	*0.061*	0.04	0.02	1.85	*0.038*
I know annual organizational goals	-0.08	0.02	-4.48	*1.000*	-0.10	0.05	-2.09	*0.977*	-0.02	0.02	-0.70	*0.754*	-0.02	0.02	-1.05	*0.848*
I know annual organizational accomplishments	0.02	0.02	1.08	*0.144*	0.07	0.05	1.40	*0.086*	0.00	0.02	-0.12	*0.549*	-0.01	0.02	-0.50	*0.689*
The training activities offered by my organizations are useful in enhancing my competences	0.17	0.02	11.20	*0.000*	0.06	0.03	1.81	*0.040*	0.13	0.02	8.42	*0.000*	0.13	0.02	8.14	*0.000*
The training activities offered by my organizations are useful in improving my communication skills with colleagues	-0.03	0.01	-2.66	*0.994*	-0.04	0.03	-1.74	*0.953*	-0.05	0.01	-4.47	*1.000*	-0.07	0.01	-5.42	*1.000*
I appreciate how managers manage the organization	1.20	0.02	57.06	*0.000*	1.01	0.05	21.39	*0.000*	1.23	0.02	53.17	*0.000*	1.22	0.02	51.75	*0.000*
My organization stimulates me to give my best in my work	0.66	0.02	27.04	*0.000*	0.98	0.05	17.88	*0.000*	0.85	0.03	32.74	*0.000*	0.87	0.03	32.88	*0.000*
I am motivated to achieve organizational goals	0.36	0.02	18.83	*0.000*	0.47	0.04	10.74	*0.000*	0.38	0.02	17.72	*0.000*	0.53	0.02	24.73	*0.000*
In my organization, merit is a fundamental value	0.02	0.02	0.78	*0.221*	-0.02	0.06	-0.36	*0.640*	0.01	0.03	0.57	*0.286*	-0.03	0.03	-1.24	*0.888*
In my organization, the professional contribution of everyone is adequately recognized	-0.07	0.02	-2.84	*0.996*	0.05	0.06	0.85	*0.201*	-0.03	0.03	-1.30	*0.897*	-0.08	0.03	-2.89	*0.996*
theta_0	4.27	0.06	66.33	*0.000*	4.39	0.16	27.97	*0.000*	4.43	0.07	62.50	*0.000*	4.30	0.07	61.60	*0.000*
theta_1	6.50	0.07	91.13	*0.000*	6.73	0.17	39.00	*0.000*	6.78	0.08	85.66	*0.000*	6.78	0.08	87.02	*0.000*
theta_2	9.30	0.08	111.11	*0.000*	9.81	0.20	48.40	*0.000*	9.70	0.09	103.71	*0.000*	9.90	0.09	106.50	*0.000*
theta_3	11.73	0.10	122.39	*0.000*	12.62	0.23	54.11	*0.000*	12.41	0.11	113.99	*0.000*	12.50	0.11	116.50	*0.000*

Table 6 presents the results of the optimization analysis for a 5 percent increase in the average value of the item “I am proud to tell others that I work in this organization.” Maintaining everything else constant, improving the mean of employees’ appreciation for how managers manage the organization is correlated to an enhanced job satisfaction at the organization level in all regions. In particular, the percentage improvement for the former statement are 12 percent for Region A, 9 percent for Region B, and 13 percent for all of the remaining regions. Furthermore, in Region B, ceteris paribus, a 9 percent percent improvement in the level of agreement with the statement that the organization stimulates employees to give their best on the job is related to the betterment of the outcome.

**Table 6:** Optimization function for employees’ satisfaction to work for their organization (+ 5 percent in the mean value)

	**Region A**	**Region B**	**Region C**	**Region D**
The equipment in my unit is adequate				
My workplace is safe (electrical systems, fire and emergency measures, etc.)				
My workload is manageable				
Meetings are organized regularly in my unit				
Work is well planned in my unit and this allows us to achieve goals				
Periodically I am given feedback from my supervisor on the quality of my work and the results achieved				
My suggestions for improvement are considered by my supervisor				
I feel like I'm part of a team that works together to achieve common goals				
My supervisor knows how to handle conflict				
I agree with the criteria adopted by my supervisor to evaluate my work				
My supervisor is fair in managing subordinates				
I believe that my supervisor carries out his job well				
My organization encourages change and innovation				
The organization encourages information sharing				
My supervisor encourages information sharing				
I know annual organizational goals				
I know annual organizational accomplishments				
The training activities offered by my organizations are useful in enhancing my competences				
The training activities offered by my organizations are useful in improving my communication skills with colleagues				
I appreciate how managers manage the organization	12%	9%	13%	13%
My organization stimulates me to give my best in my work		9%		
I am motivated to achieve organizational goals				
In my organization, merit is a fundamental value				
In my organization, the professional contribution of everyone is adequately recognized				

Table 7 displays estimates from a logistic regression model for public employees’ satisfaction to work for the health service of their regional government. Keeping everything else equal, across regions, the positive correlates at the 0.05 significance level are the following: workplace safety, supervisor’ adequate competences to carry out the job, effective training in improving one’s skills, appreciation for how managers run the organization, organizational stimuli to give one’s best on the job, and motivation to achieve organizational mission. Having an adequate equipment is positively associated with the satisfaction to work for the health care system at the standard statistical levels in all regions but C and D. The relationship between the satisfaction to work for one’s organization and the degree to which one’s work is manageable is positive at the 0.05 significance level for all regions except Region B, where the relationship is marginally significant (*p* = *0.054*). Employees’ perceptions that their suggestions for improvement are taken into consideration by their supervisors are significantly related to satisfaction in regions B, C, and D (*p* = *0.009*, *p* = *0.008*, and *p* = *0.024* respectively). Region C is the only that displays a positive correlation between the satisfaction to serve the health systems and an agreement with the criteria adopted to evaluate individual performance, ceteris paribus (*p* = *0.001*). Regions C shows a positive correlation between information sharing at the organizational level and work satisfaction (*p* = *0.003*), whereas team-level information sharing is relevant in Region D (*p* = *0.006*). Then, awareness of the organizational goals is a relevant predictor of the satisfaction to work for the health service on one’s regional government in Region D (*p* = *0.006*).

**Table 7:** Logistic regression results for employees’ satisfaction to work for the health service of their Regional government

	**Region A**				**Region B**				**Region C**				**Region D**			
	**b**	**SE**	**z**	***p***	**b**	**SE**	**z**	***p***	**b**	**SE**	**z**	***p***	**b**	**SE**	**z**	***p***
The equipment in my unit is adequate	0.07	0.01	5.13	*0.000*	0.07	0.03	2.35	*0.013*	0.02	0.01	1.17	*0.127*	0.00	0.01	0.11	*0.455*
My workplace is safe (electrical systems, fire and emergency measures, etc.)	0.12	0.01	9.28	*0.000*	0.19	0.03	6.45	*0.000*	0.09	0.01	7.20	*0.000*	0.11	0.01	8.23	*0.000*
My workload is manageable	0.08	0.01	6.55	*0.000*	0.04	0.03	1.66	*0.054*	0.09	0.01	7.40	*0.000*	0.08	0.01	6.53	*0.000*
Meetings are organized regularly in my unit	-0.05	0.01	-4.52	*1.000*	-0.04	0.03	-1.50	*0.928*	-0.06	0.01	-5.13	*1.000*	-0.01	0.01	-1.04	*0.846*
Work is well planned in my unit and this allows us to achieve goals	0.02	0.02	1.29	*0.104*	0.04	0.04	1.13	*0.135*	0.01	0.02	0.53	*0.300*	-0.02	0.02	-1.06	*0.850*
Periodically I am given feedback from my supervisor on the quality of my work and the results achieved	-0.07	0.01	-5.13	*1.000*	-0.09	0.03	-2.76	*0.995*	-0.09	0.02	-5.40	*1.000*	-0.13	0.02	-7.91	*1.000*
My suggestions for improvement are considered by my supervisor	0.02	0.02	1.24	*0.113*	0.09	0.04	2.54	*0.009*	0.04	0.02	2.57	*0.008*	0.04	0.02	2.06	*0.024*
I feel like I'm part of a team that works together to achieve common goals	0.13	0.01	8.91	*0.000*	0.16	0.03	4.75	*0.000*	0.12	0.02	7.49	*0.000*	0.15	0.02	9.16	*0.000*
My supervisor knows how to handle conflict	-0.10	0.02	-5.49	*1.000*	-0.08	0.04	-1.90	*0.966*	-0.08	0.02	-4.04	*1.000*	-0.07	0.02	-3.43	*0.999*
I agree with the criteria adopted by my supervisor to evaluate my work	0.03	0.02	1.44	*0.080*	-0.03	0.04	-0.84	*0.796*	0.07	0.02	3.64	*0.001*	0.03	0.02	1.39	*0.087*
My supervisor is fair in managing subordinates	-0.04	0.02	-2.38	*0.988*	-0.11	0.04	-2.78	*0.995*	-0.07	0.02	-3.92	*1.000*	-0.03	0.02	-1.41	*0.915*
I believe that my supervisor carries out his job well	0.12	0.02	5.95	*0.000*	0.12	0.05	2.65	*0.007*	0.10	0.02	4.63	*0.000*	0.07	0.02	3.25	*0.002*
My organization encourages change and innovation	-0.02	0.02	-1.14	*0.867*	-0.01	0.04	-0.36	*0.639*	0.01	0.02	0.61	*0.274*	0.03	0.02	1.46	*0.078*
The organization encourages information sharing	0.02	0.02	1.47	*0.077*	0.00	0.03	0.13	*0.448*	0.05	0.02	3.03	*0.003*	0.02	0.02	1.16	*0.129*
My supervisor encourages information sharing	0.02	0.02	1.36	*0.092*	0.04	0.04	1.09	*0.142*	0.02	0.02	1.04	*0.154*	0.05	0.02	2.66	*0.006*
I know annual organizational goals	-0.01	0.02	-0.49	*0.685*	-0.06	0.04	-1.47	*0.924*	0.01	0.02	0.50	*0.310*	0.06	0.02	2.68	*0.006*
I know annual organizational accomplishments	0.00	0.02	0.07	*0.471*	0.02	0.04	0.52	*0.305*	0.00	0.02	-0.10	*0.540*	-0.07	0.02	-3.09	*0.998*
The training activities offered by my organizations are useful in enhancing my competences	0.23	0.01	15.68	*0.000*	0.18	0.03	5.86	*0.000*	0.22	0.01	14.81	*0.000*	0.25	0.02	16.31	*0.000*
The training activities offered by my organizations are useful in improving my communication skills with colleagues	-0.04	0.01	-3.81	*1.000*	-0.04	0.02	-1.87	*0.964*	-0.05	0.01	-3.96	*1.000*	-0.07	0.01	-5.94	*1.000*
I appreciate how managers manage the organization	0.45	0.02	24.47	*0.000*	0.35	0.04	8.25	*0.000*	0.46	0.02	22.90	*0.000*	0.39	0.02	19.06	*0.000*
My organization stimulates me to give my best in my work	0.23	0.02	10.24	*0.000*	0.33	0.05	6.67	*0.000*	0.29	0.02	12.33	*0.000*	0.26	0.02	11.00	*0.000*
I am motivated to achieve organizational goals	0.47	0.02	25.13	*0.000*	0.51	0.04	12.38	*0.000*	0.45	0.02	22.33	*0.000*	0.55	0.02	27.25	*0.000*
In my organization, merit is a fundamental value	-0.04	0.02	-1.52	*0.930*	0.02	0.05	0.32	*0.376*	-0.05	0.02	-2.11	*0.978*	-0.06	0.02	-2.50	*0.991*
In my organization, the professional contribution of everyone is adequately recognized	-0.06	0.02	-2.60	*0.993*	-0.04	0.05	-0.69	*0.751*	-0.03	0.02	-1.22	*0.884*	-0.06	0.02	-2.47	*0.990*
theta_0	1.44	0.06	25.17	*0.000*	1.38	0.14	9.84	*0.000*	1.43	0.06	23.13	*0.000*	1.14	0.06	18.44	*0.000*
theta_1	2.99	0.06	51.60	*0.000*	2.98	0.14	21.26	*0.000*	2.97	0.06	47.58	*0.000*	2.69	0.06	43.94	*0.000*
theta_2	5.11	0.06	80.17	*0.000*	5.19	0.15	33.99	*0.000*	5.04	0.07	73.64	*0.000*	4.86	0.07	72.48	*0.000*
theta_3	6.82	0.07	98.22	*0.000*	7.04	0.17	42.54	*0.000*	6.78	0.07	90.82	*0.000*	6.55	0.07	89.90	*0.000*

Similarly, to Tables 3 and 6, Table 8 displays the findings from an optimization algorithm aimed at improving the mean value of the satisfaction to work for the health service of one’s regional government by 5 percent. Improving positive perceptions about how managers run the organization and the motivation to achieve the organizational mission are correlated to an enhanced job satisfaction. In particular, the percentage improvement for the former statement are 11 percent for Region A, 7 percent for Region B, 13 percent for Region C, and 7 percent for Region D. As to the latter, the percentages are, respectively; 12, 15, 12, and 15. In Regions A and D, improving by 1 percent and 2 percent the mean value associated with the usefulness of training for competence enhancement are linked to a higher satisfaction. In Region B, instead, an improvement of the 6 percent of the organizational stimuli to give the best in one’s work correlated with an increased satisfaction. Lastly, improving personnel’s perceptions about workplace settings by 1 percent is associated with a higher satisfaction in Region B.

**Table 8:** Optimization function for employees’ satisfaction to work for the health service of their Regional government (+ 5 percent in the mean value)

	**Region A**	**Region B**	**Region C**	**Region D**
The equipment in my unit is adequate				
My workplace is safe (electrical systems, fire and emergency measures, etc.)		1%		
My workload is manageable				
Meetings are organized regularly in my unit				
Work is well planned in my unit and this allows us to achieve goals				
Periodically I am given feedback from my supervisor on the quality of my work and the results achieved				
My suggestions for improvement are considered by my supervisor				
I feel like I'm part of a team that works together to achieve common goals				
My supervisor knows how to handle conflict				
I agree with the criteria adopted by my supervisor to evaluate my work				
My supervisor is fair in managing subordinates				
I believe that my supervisor carries out his job well				
My organization encourages change and innovation				
The organization encourages information sharing				
My supervisor encourages information sharing				
I know annual organizational goals				
I know annual organizational accomplishments				
The training activities offered by my organizations are useful in enhancing my competences	1%			2%
The training activities offered by my organizations are useful in improving my communication skills with colleagues				
I appreciate how managers manage the organization	11%	7%	13%	7%
My organization stimulates me to give my best in my work		6%		
I am motivated to achieve organizational goals	12%	15%	12%	15%
In my organization, merit is a fundamental value				
In my organization, the professional contribution of everyone is adequately recognized				

Overall, our analyses present three main key findings. First, within dependent variables, the correlates of job satisfaction tend to be the same across the health services of four regional governments. Second, the correlates of job satisfaction seem to differ among outcomes, namely hierarchical level at which employees’ satisfaction is measured. Third, context-specific associations emerge from our models.

## Discussion

Our work aimed at (i) investigating the correlates of health professionals’ job satisfaction at three hierarchical levels, namely satisfaction to work for one’s unit, organization, and health system of the regional government, and (ii) predicting how the improvement of the average value of correlates may relate with the improvement in the outcome variables. We employed large-scales observational surveys across healthcare systems in Italy. A series of logistic regressions reveal that environmental characteristics, management practices at the organizational level, and management practices at the team level correlates with work satisfaction. The pattern of results seems to replicate across outcome variables and healthcare systems. A series of optimization algorithms show that improving how the work is organized at the unit level, the degree to which employees perceive a sense of being part of a team with shared goals, and the supervisor’s abilities in carrying out the job may correlate with a better satisfaction to work for one’s unit. To the contrary, improving how managers perform their job tend to be associated with more satisfaction to work for one’s organization. As to the satisfaction to serve one’s regional health system, then, an improved work satisfaction correlates with an improved appreciation for the top management and the motivation to achieve the organizational mission.

The correlates that may relate to a higher job satisfaction are, therefore, in part different among hierarchical levels [2, 18, 52]. Within outcome variables, the largest variation in the correlates of job satisfaction is to the regional government level. Taken together, these findings align with two well established literature streams. On the one hand, attitudes and needs are so deeply seated in the human nature that they tend to be invariant for work satisfaction at the micro-level [8, 43]. On the other hand, then, characteristics contingent to the macro-level may be relevant in prioritizing some attitudes and needs over others [6, 9, 16].

Further on the previous point, our work seems to suggest that all governance levels can play a role in employees’ job satisfaction, which continues to be a topic of interest for research syntheses attempts at the international level [53–55]. Some of the levers may overlap whereas other are different. As to the former, for instance, the quality and competence of managers at the unit and organizational level both correlated with work satisfaction. Thus, the mix of levers and the extent to which they are used may vary across regional healthcare system, which ultimately represent the highest governance level. Research on this consideration seems to have become even more prominent in the wake of the COVID-19 pandemic [56].

Our study may provide a few contributions to extant scholarship and practice on job satisfaction in public service. Firstly, we investigate the correlates of satisfaction at three hierarchical levels. To the best of our knowledge, while most research analyzed satisfaction using hierarchical models [9], they tend to focus on one level only. Secondly, our analyses tap into many correlates of job satisfaction. This has the potential to uncover unexpected associations. Routine and large-scale survey on public employees’ perceptions provide a natural opportunity to engage in broad and deep understanding of organizational phenomena in the management of human resources. Thirdly, we introduce optimization models as a way to provide practitioners-friendly predictions on combinations of job satisfaction constructs that may be worth considering together to improve well-being. We are not aware of any such approach as far as managing public personnel is concerned. Fourthly, unlike most scholarship, our work is based on large-sample surveys and replication efforts aimed at the testing the generalizability of the findings.

## Limitations

From a practitioner standpoint, the main limitation of our study is that it provides valuable insights targeted to decision makers at the regional level. In other words, it is beyond the scope of this investigation providing analyses at the organizational level. The degree to which findings aggregated by region generalize to results aggregated by organization within regions remains to be tested. Similarly, providing analysis across typologies of health professionals – also through customized survey instruments – is outside the scope of our work, though an avenue of future work that might be worth pursuing.

Then, we must acknowledge that our work suffers from the same limitations that affect observational studies and combine logistic regression analyses with optimization techniques. Most notably, we are unable to establish cause-effect relationships between job satisfaction and its determinants or consequences. As to the representativeness of the sample, the inability to compare demographic statistics between the sample and the exact population of reference is due to the general data protection regulation—defined at the European Union level and detailed in national states—that is fully binding when doing research with real organizations. The regulation prohibits analyzing variables before the data collection is closed and storing any information of non-respondents. Although, a response rate of 80% or more is desired to establish scientific validity in epidemiology, researchers demonstrated that reaching that response rate is not always possible and can lead to other problems [57]. In addition, the response rates in our samples appear to be in line with those of established surveys, such as the NHS survey – where the lates response rate reached 46% or the Federal Employee Viewpoint Survey – which registered a 34% participation in the latest edition. Of course, readers are encouraged to always keep in mind this feature when considering our work. Furthermore, concerns about the generalizability of results across operations (importantly of the job satisfaction variables), settings, and samples are legitimate. Similarly, the generalizability of our findings from the optimization analyses to other healthcare systems around the world is unknown because, to the best of our knowledge, this has no prior in the literature. Unfortunately, we are unable, at the moment, to expand our work by adding data collected in other countries around the globe. We very much encourage replication studies, which would serve as rigorous and challenging external validity tests of the current work. In fact, replication efforts are common practice for other topics in the healthcare management domains. As to regression analyses, omitted variable biases may impinge on the validity of the findings. Moreover, our analyses are nested within regions and comparisons across regions must be done with caution. In fact, our logistic regressions do not account for variables such as socio-demographic items that may be distributed differently in different regional healthcare systems.

As to the optimization techniques, we acknowledge that its sensitivity to changes in the magnitude of regression coefficients and the lack of cost structure impose a warning in deriving implications for practice. Indeed, the optimization model selects the best combination of correlates that might associate with an improved outcome based on their mean value and relative strength. This influences the stability of the optimization results. Also, the algorithm identifies a set of factors that together generate a preset level of increase in the overall satisfaction measures. Although these results are optimal within the context in which they were presented, they may not be the best possible from a cost perspective. Lacking cost information, the algorithm assumes that the cost to improve each of the predictors is equivalent. Form a practical perspective, however, implementing changes suggested by our findings may not translate into the most cost-effective reforms. To the contrary, there might be other interventions that improve job satisfaction and are less costly.

## Conclusion

Our work on the job satisfaction correlates of about 73,000 public health employees paves the way for a more extensive use of work satisfaction and organizational climate survey among typologies of mission-driven organizations. Whereas questionnaires measuring the attitudes and the perceptions of government personnel such as the Federal Employee Viewpoint in the United States or of health professionals such as the survey of National Health System in the United Kingdom are now spread around the globe, similar inquiry are not yet common practice in other public institutions. Our study may be a systematic attempt to fill this gap. Furthermore, we emphasize the need to use any such survey for managerial efforts aimed at improving the quality of the organization and the well-being of their employees. In this regard, the optimization model seems helpful in deriving implications for practice.

## Data Availability

The datasets used and/or analysed during the current study are not publicly available to maintain employers' and employees' confidentiality but are available from the corresponding author on reasonable request.
